# EPC infusion ameliorates acute graft-versus-host disease-related endothelial injury after allogeneic bone marrow transplantation

**DOI:** 10.3389/fimmu.2022.1019657

**Published:** 2022-12-14

**Authors:** Weiwei Wang, Yali Ye, Yuwei Du, Zhengqing Xu, Ke Yuan, Yizhou Wang, Seyram Yao Adzraku, Yue Li, Kailin Xu, Jianlin Qiao, Wen Ju, Lingyu Zeng

**Affiliations:** ^1^ Blood Diseases Institute, Xuzhou Medical University, Xuzhou, Jiangsu, China; ^2^ Department of Hematology, The Affiliated Hospital of Xuzhou Medical University, Xuzhou, Jiangsu, China; ^3^ Jiangsu Key Laboratory of Bone Marrow Stem Cells, Xuzhou, Jiangsu, China

**Keywords:** endothelial progenitor cell, acute graft-versus-host disease, endothelium, endothelial injury, endothelial cell activation

## Abstract

**Introduction:**

Graft-versus-host disease (GVHD) damages vascular endothelium. Endothelial progenitor cell (EPC) can differentiate to endothelial cell and promote angiogenesis, but its role in endothelial damage in GVHD is unclear.

**Methods:**

In this study, we intend to assess whether EPC infusion promotes the repair of endothelial injury in GVHD mouse model. Male BALB/c mice were randomly divided into 5 groups: control group, total body irradiation group (TBI group), allogeneic bone marrow transplantation group (Allo-BMT group), acute graft versus host disease group (GVHD group), EPC infusion group (GVHD+EPC group) followed by analysis of mice survival, acute GVHD (aGVHD) score, T cell infiltration by immunofluorescence, as well as continuity of vascular endothelium in liver.

**Results:**

Compared with Allo-BMT group, the clinical and pathological score of aGVHD mice were higher. On day 21 after transplantation, a large number of mononuclear cell infiltrations were seen in the target tissues of aGVHD mice and mice died within 30 days. In addition, aGVHD group also presented increased subendothelial infiltration of CD3^+^ T cells in the liver, decreased VE-cadherin expression and elevated major histocompatibility complex (MHC) II molecule expression in the endothelium. Moreover, expression of MHC-II molecule increased in endothelial cell after irradiation injury and LPS stimulation, indicating abnormally activated endothelial cell with antigen-presenting function. Interestingly, infusion of EPC reduced the clinical and pathological score of aGVHD, decreased infiltration of mononuclear cells, improved survival as well as upregulated VE-cadherin and downregulated MHC-II molecule.

**Discussion:**

EPC infusion can mobilize to affected endothelium to decrease the infiltration of T cells and pathological endothelial activation contributing to ameliorating the damage of endothelium. EPC infusion combined with bone marrow transplantation might be a perspective strategy for the prevention and treatment of aGVHD.

## Introduction

Allogeneic bone marrow transplantation (allo-BMT) is a clinically effective method for treating refractory blood diseases (1). Graft-versus-host disease (GVHD) is one of the main complications after allo-BMT and restricts the transplantation efficacy (2). According to statistics, the incidence of acute GVHD (aGVHD) after transplantation ranges from 54% to 62% (3), and the rate of overall survival for 5 years is 46% (4). Therefore, GVHD prevention and treatment are urgently required to be further explored.

aGVHD is an inflammatory disease that mainly affects the liver, skin, and intestines. Since these target organs are affected generally by endothelial dysfunction and the proposal of endothelium-related aGVHD provides new ideas and therapeutic targets for the treatment, and is a research hotspot in recent years (5, 6). Endothelial cells are the first barrier for bacteria and viruses in the blood to enter the tissues. They participate in maintaining the blood homeostasis of the body, including regulation of vascular integrity and host defense. Endothelial injury and dysfunction have an important impact on the health of the body. At the same time, endothelial cell can act as antigen presenting cell. Under the stimulation of inflammatory factor, endothelial cell highly expresses the key molecules of antigen presentation and co-stimulation such as MHC-II molecule, CD40 and ICOSL, participates in antigen presentation and promotes T cell activation (7, 8). Studies have showed that in allo-BMT, the fragility and dysfunction of endothelial cell (9) is not only a cause of refractory GVHD (10), but also an important factor in delaying the recovery of organ function (11, 12). Therefore, vascular endothelium is a potential target for the prevention and treatment of GVHD.

Endothelial progenitor cell (EPC) is the precursor cell of endothelial cell, derived from bone marrow, fat, spleen and other tissues, and plays an important role in maintaining endothelial integrity of blood vessels and repairing endothelial damage (13, 14). EPC can mobilize to the damaged blood vessel and differentiate into mature endothelial cell. EPC therapy has been considered as a promising treatment strategy in the fields of nephropathy and cerebral ischemic diseases (15, 16). Huang X et al. have reported that infusion of EPC can reverse the cerebral vascular damage caused by irradiation possibly through increasing the expression of tight junction proteins in the brain and reducing the permeability of the blood-brain barrier, which may restore the effect of whole brain irradiation on the blood brain damage (15). Our previous research found that infusion of EPCs can alleviate aGVHD and enhance immune reconstitution after bone marrow transplantation (17), but the mechanism is unclear. Therefore, in-depth study of endothelial damage in aGVHD and the mechanism of EPC in repairing the endothelium is the key to explore strategies to alleviate aGVHD. Our study intends to assess the effects of EPCs infusion on damaged endothelium in aGVHD mouse model to explore new strategies for the prevention and treatment of aGVHD.

## Materials and methods

### Reagents

Rabbit anti-mouse GFP antibody, Rabbit anti-mouse CD3 antibody and goat anti-rabbit AF488 were purchased from abcam; rat anti-mouse CD31, CD144 and MHC-II molecule monoclonal antibody were purchased from eBioscience; goat anti-rat or anti-rabbit Cy3, anti-fluorescence quenching agent and rabbit anti-mouse CD31 polyclonal antibody were purchased from Servicebio; goat anti-rat AF488 was purchased from CST; EGM-2 medium was purchased from Lonza; Fibronectin was purchased from EMD Millipore; Anti-Mouse-CD31-V450, anti-Mouse-VEGFR2-PE and anti-Mouse-CD45-PerCP Cy7 were bought from BD; 7-AAD was purchased from Nanjing KGI Biotech Co., Ltd.; mouse endothelial cell growth factor (VEGF) was bought from Proteintech; Accutase-Enzyme Cell Detachment Medium was purchased from Thermo Fisher Company.

### Experimental animals

SPF-grade BALB/c (H-2kd) and C57BL/6 (H-2Kb) mice weighted 18-20 g and aged 6-8 weeks were purchased from Beijing Vitality China Laboratory Animal Co., Ltd. B6.Cg-Tg (CAG-GFP) mice were purchased from Shanghai Model Organisms Center (China). The mice were fed in the experimental animal center with free access to food and water. This study was approved by the Animal Ethics Committee of Xuzhou Medical University.

### Preparation of bone marrow cell

Wild-type C57BL/6 female mice were sacrificed by cervical dislocation and soaked in 75% alcohol for 5 minutes; sterile scissors and tweezers were used to remove the tibia, femur and ilium which were then immersed in a dish filled with PBS. Sterile gauze was used to remove the muscle tissue and fascia from the bone and then placed in a mortar followed by gentle crushing. Next, an appropriate amount of PBS was added to disperse cells as much as possible. The bone marrow suspension was filtered, centrifuged at 1200rpm for 5min, washed once with PBS, counted, and then diluted to 2.5x10^7^/mL for later use.

### Preparation of splenic mononuclear cell

After mice were sacrificed by cervical dislocation, they were immersed in 75% ethanol for 5 minutes for disinfection. The mouse spleens were taken out on a clean bench and placed in a pre-prepared dish containing PBS for later use. 1 mL of lymphocyte separation solution (dakeve) was added and the spleen was grinded followed by addition of 4 mL of lymphocyte separation solution to obtain a single cell suspension, which was filtered with a 200 mesh filter and centrifuged at 800 g for 30 min to obtain spleen lymphocyte suspension. Then, the cell suspension was counted and adjusted to 2.5x10^7^/mL for later use.

### Preparation of EPC

Bone marrow mononuclear cells in EGM-2 complete medium (5×10^6^/ml) were placed into culture dish coated with fibronectin (FN). The medium was changed every 3 days. When being cultured to the 7th day, the cells were identified by flow cytometry (FACS). The identification index was: CD45^-^CD31^+^ VEGFR2^+^. Cell viability was assessed using 0.4% trypan blue dye solution and the viability above 95% was used for experiments.

### aGVHD model

BABL/c male mice of 6-8 weeks old were randomly divided into 5 groups. Untreated group: normal BALB/c mice without any treatment; total body irradiation group (TBI group): The mice were fed with sterilized water contained a moderate dose of antibiotics one week before irradiation. The mice received total body irradiation using Cs137caesium gamma irradiator with a total dose of 7.5Gy. After 6 hours, 0.25 mL of PBS was infused through the tail vein. Allo-BMT group: On the basis of TBI group, 0.25 mL of cell suspension containing C57BL/6 mouse-derived bone marrow mononuclear cells (5×10^6^ cells) were infused *via* the tail vein; GVHD group: on the basis of TBI group, infusion of 0.25 mL of cell suspension containing C57BL/6 mouse-derived bone marrow mononuclear cells (5×10^6^ cells) and splenic mononuclear cells (5×10^6^ cells); GVHD+EPC group: On the basis of TBI, infusion of 0.25 mL of cell suspension containing bone marrow mononuclear cells (5×10^6^ cells), splenic mononuclear cells (5×10^6^ cells) and Bone marrow-derived EPCs (5×10^5^ cells).

### Clinical aGVHD score

One day before irradiation and every three days after transplantation, weight, appearance and comprehensive movement of mice were observed. GVHD was evaluated according to the Cooke scoring system (18). The clinical scoring criteria for aGVHD include weight loss, posture, mobility, fur changes and skin integrity. According to the criteria, a score of 0-2 is given daily to each mouse for each criteria and all the individual scores were summed as the total score of each mouse. The data of each group were obtained from 6 mice.

### GVHD pathology score

After transplantation, the liver, ileum and colon of each group were obtained and fixed in 4% paraformaldehyde for 48h followed by dehydration with gradient alcohol, embedding, sectioning, and H&E staining. Acute GVHD score was analyzed under a microscope according to GVHD pathological scoring standard (19, 20). Simply, Ileum: villous blunting, loss of enterocyte brush border, crypt regeneration, crypt cell apoptosis and crypt destruction, lamina propria lymphocytic infiltrate; colon: colonocyte vacuolization and surface colonocyte attenuation, crypt regeneration, crypt cell apoptosis and crypt destruction, lamina propria lymphocytic infiltrate. Liver: subendothelial infiltrate of mononuclear cells in each section. The scoring system denoted 0: normal; 1.0: focal and rare; 2.0: diffuse and mild; 3.0: diffuse and moderate; 4.0: diffuse and severe. All of each score were added to obtain a total score for each specimen. The data of each group were obtained from 4-6 mice.

### Cell tracking assay

BABL/c male mice of 6-8 weeks old were infused with 5 × 10^5^ EPCs derived from B6.Cg-Tg (CAG-GFP) mice and 5×10^6^ bone marrow mononuclear cells after lethal dose irradiation. At week 4 post infusion, the liver and intestine of infused mice (n=3) were obtained and tested by frozen section and immunofluorescent staining.

### Immunofluorescence staining

At the proposed time point after transplantation, the liver, ileum, and colon of each group of mice (n=3-5) were obtained, dehydrated, embedded and sliced into 7-9μm thickness by RM2126 microtome. The sections were fixed in pure methanol pre-cooled at -20°C, blocked in 5% BSA-PBS solution, and incubated with primary antibodies against CD31, CD3, CD144, MHC-II, or CD31, GFP. Then, it was incubated with secondary antibodies (rabbit anti-rat Cy3, goat anti-rabbit Cy3, goat anti-rabbit AF488, goat anti-rat AF488). After that, nucleus was stained with DAPI. The slide was mounted and observed under a confocal microscope (Zeiss 880).

### Endothelial cell activation

bEnd.3 cells (brain-derived Endothelial cells.3, purchased from ATCC) (1 × 10^5^ per well in 6-well plate) were injured by irradiation of gamma irradiator at a dose of 15 Gy followed by adding LPS (Sigma, 100 ng/ml) into cell culture supernatant. The control cells were cultured with no irradiation or LPS. After 24 hours of culture, the cells of two groups were harvested for immunofluorescence staining.

### Statistical processing

Mice or cells were allocated to experiments randomly and samples were processed in an arbitrary order. GraphPad Prism 8.0 software was used for analyzing all data which were expressed as mean ± SEM and evaluated by t-test or a one-way ANOVA. *p*<0.05 indicates a difference.

## Results

### EPC infusion in combination with allogeneic bone marrow transplantation reduces GVHD severity

**Figure 1:**
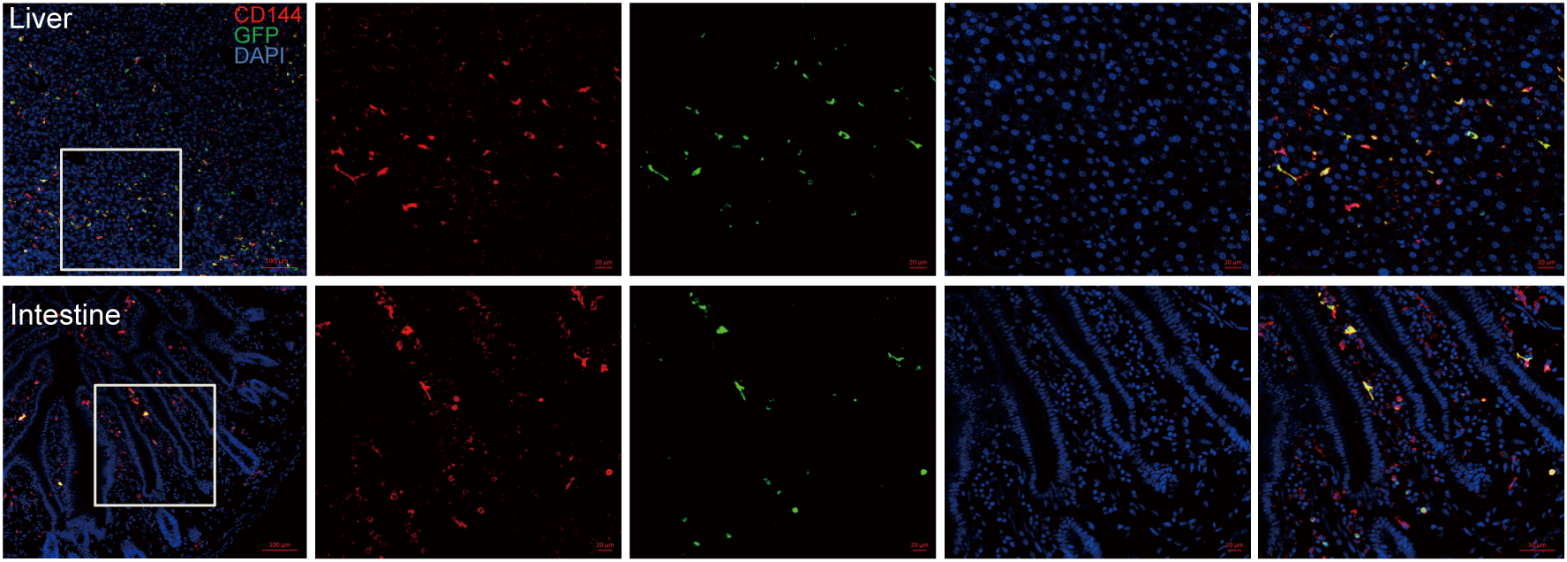
EPC homing to mouse liver and intestinal vascular endothelium after infusion. BALB/c mice were irradiated for lethal dose and infused with C57BL/6 mouse-derived bone marrow mononuclear cells (5×10^6^ cells) and EPCs (5×10^5^ cells) derived from B6.Cg-Tg (CAG-GFP) mice, and liver and intestine of the mice were obtained after 4 weeks post-transplantation for dehydration, embedding, and frozen section followed by immunofluorescence staining by antibodies against mice CD144 and GFP. Red: CD144, green: GFP, blue: nucleus. n=3. Upper: liver; lower: intestine.

We assessed whether EPCs could home to liver and intestinal tissues by infusion of GFP-expressing EPCs (derived from B6.Cg-Tg (CAG-GFP) mice). After 4 weeks post-infusion, GFP^+^ cells were still found in liver and intestinal sections of mice and co-localized with the endothelial-specific marker CD144 (**Figure 1**), suggesting that EPCs could home to liver and intestine and differentiate to endothelial cells.

**Figure 2:**
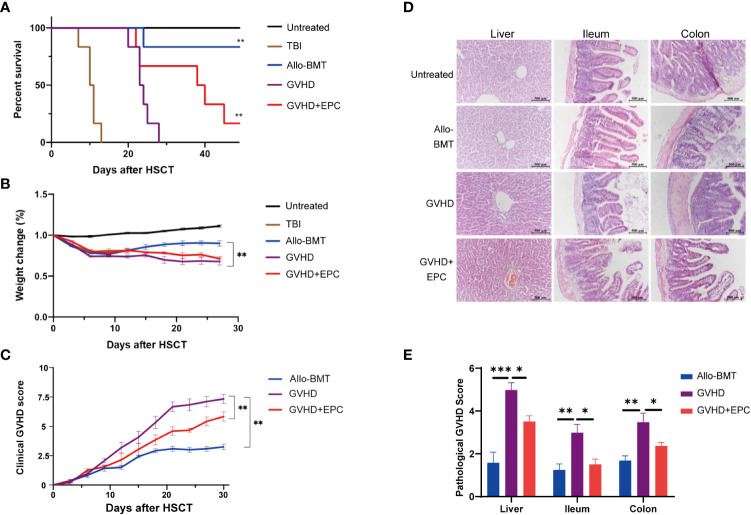
Comparison of survival time, clinical scores and histopathological scores of mice in each group after transplantation. All mice were observed for The survival curve **(A)**, Weight change **(B)** and Clinical score of GVHD **(C)** every three days after transplantation (n=6, **P<0.01). On day 21 after transplantation, liver, ileum, and colon of mice were obtained for dehydration, embedding, sectioning and H&E staining [×200, **(D)**]. **(E)** The pathological scores of sections were evaluated by a light microscope according GVHD pathological scoring standard ^19,20^ and analyzed by GraphPad Prism 8.0 software. (n=4-6, *P<0.05, **P<0.01, ***P<0.001).

The mice in untreated group had smooth and dense hair, agile and free movement, intact skin, ruddy mucous membranes without abnormalities in diet, urine or feces. Three days after irradiation, mice in TBI group showed signs of back curling, bristles, and unsatisfactory hair after irradiation. After 7 days, they showed obvious listlessness, reduced food intake, decreased mobility and died on the 7th day. The mice in Allo-BMT group showed irradiated changes such as dull skin and hair, but no obvious symptoms such as arched back, diarrhea, and decreased mobility. Compared with Allo-BMT group, mice of GVHD group showed severe clinical symptoms and high mortality. One week after transplantation, mice in GVHD group presented scattered fur, lack of energy, reduced food intake, and gradually developed hair loss, diarrhea, hunched back, and significantly decreased mobility. 21 days after transplantation, the clinical score of GVHD group was significantly higher than that of Allo-BMT group (P<0.01). However, the survival rate of GVHD+EPC group was increased to 65.5% at 30 days and 32.2% at 40 days after transplantation (**Figures 2A**). Compared with GVHD group, the GVHD+EPC group had a lower clinical score (P<0.01) (**Figures 2B, C**). This data showed that EPC infusion could alleviate GVHD and prolong the survival time of mice.

In addition, liver and intestine were collected on day 21 after transplantation for pathological analysis. The results indicated that compared with Allo-BMT, GVHD mice showed significantly more inflammatory cell infiltration in the central vein and venules of the liver and the intestinal mucosa, dilation and congestion of central vein and hepatic sinuses, edema in mucosa and submucosa. Meanwhile, the pathological scores of liver and intestinal tissues in GVHD group were significantly higher than those in Allo-BMT group (p<0.05). After EPC infusion, the infiltration of mononuclear cells in the liver and intestinal mucosa of mice was reduced with more consecutive vascular morphology in the liver and increased goblet cells in the intestinal tissue, the pathological scores of liver and intestinal tissues were lower significantly compared with GVHD group (P<0.05) (**Figures 2D, E**). Taken together, co-infusion of EPC could reduce the pathological damages of GVHD targeted tissues. To explore the underlying mechanism of EPC on T cell subsets which play key role in the pathogenesis of GVHD, we analyzed levels of effector T cell subsets in mice on day 21 after transplantation. Compared with GVHD group, proportions of Th1 and Th17 cells, which are implicated inflammatory T cell subtypes inducing GVHD, did not change significantly in GVHD+EPC group. However, proportions of Th2 and Treg cells, which have been found to play protective role in GVHD, increased significantly in GVHD+EPC group (**Supplemental Figure 1**). It suggested that EPC infusion modulates effector T cell subsets contributing to reducing aGVHD severity.

### Infusion of EPC reduces T cell infiltration in the tested organs of GVHD

**Figure 3:**
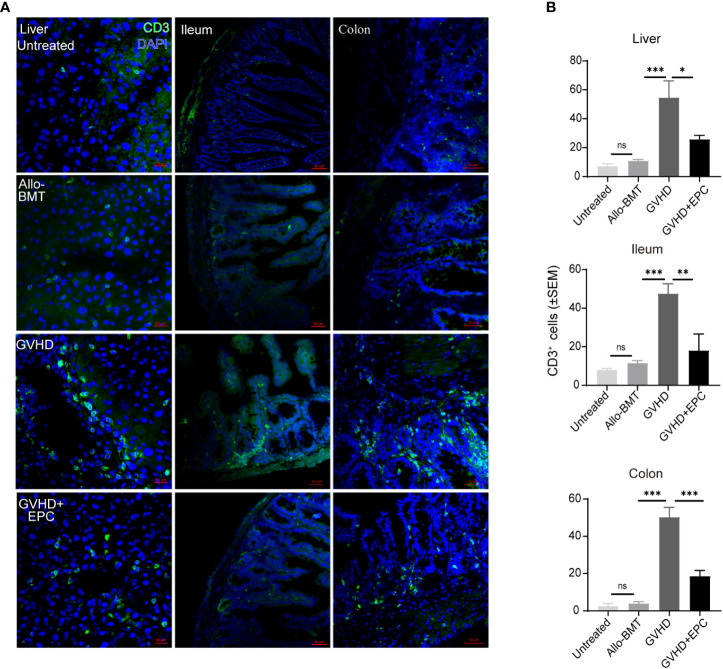
CD3^+^ T cell infiltration in the liver, ileum or colon tissue. On day 21 after transplantation, livers, ileums, and colons of each group of mice were collected for dehydration, embedding, and frozen section followed by immunofluorescence staining using antibodies against mice CD3 **(A)** and quantitative analysis the means of CD3^+^ T cells under a view by randomly 5 views of livers, ileums, and colons **(B)** (n=4-5, *P<0.05, **P<0.01, ***P<0.001). ns, no significance.

T lymphocyte infiltration was correlated with the pathological grade of GVHD. Therefore, we test T cell infiltration of liver, ileum and colon in aGVHD mice and the effect of infusion of EPC combined with bone marrow. On day 21 after transplantation, liver, ileum and colon were tested for immunofluorescence staining of T cells. As shown in **Figure 3**, GVHD group mice showed a large number of CD3^+^ T cells infiltrated around the central vein and venules of the liver, in the submucosa of the ileum and colon with a significant difference compared with BMT group (P<0.01). However, the number of CD3^+^ T cells infiltration in livers, ileum and colon was significantly reduced in GVHD+EPC group (P <0.05) (**Figure 3**).

### Infusion of EPC ameliorates the damage of endothelium in the liver

**Figure 4:**
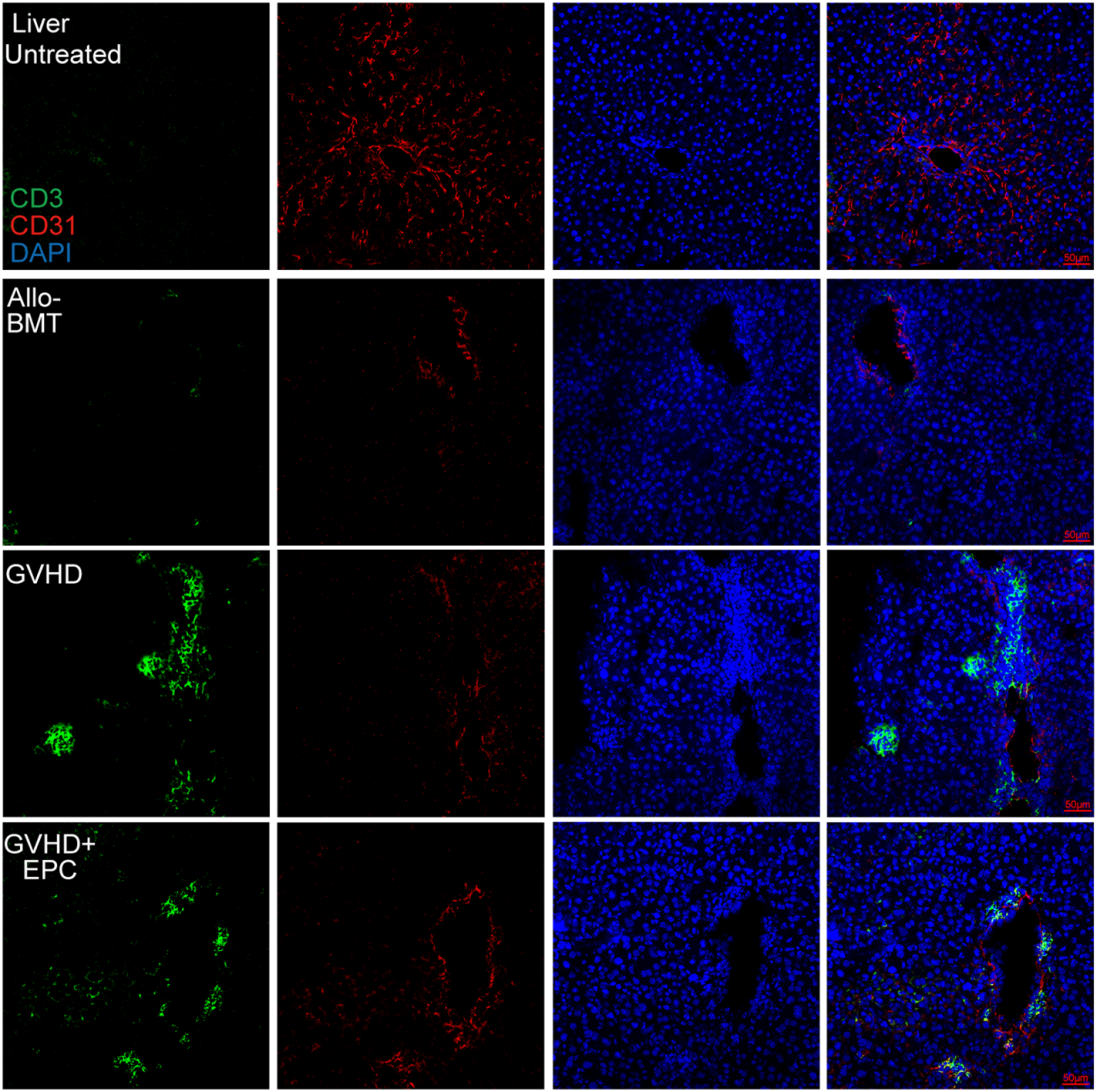
CD3^+^ T cell infiltration related to endothelium in the liver. On day 21 after transplantation, livers of each group of mice were obtained for dehydration, embedding, and frozen section followed by immunofluorescence staining using antibodies against mice CD3 and CD31. Red shows CD31-labeled vascular endothelium, green shows CD3^+^ T cells, and blue shows nuclei.

**Figure 5:**
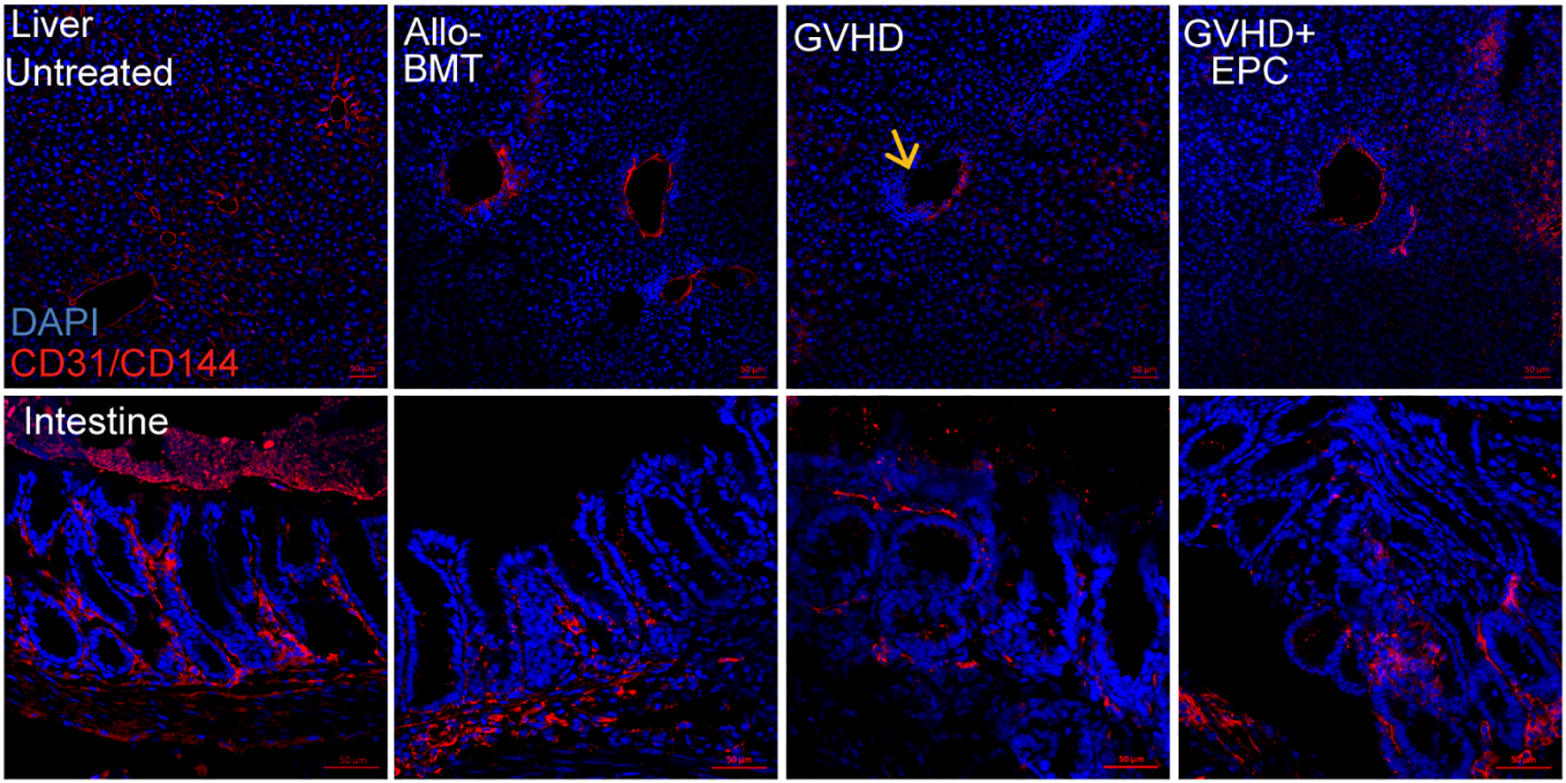
VE-cadherin/CD31 expression in liver tissue. On day 21 after transplantation, livers of each group of mice were obtained for dehydration, embedding, and frozen section followed by immunofluorescence staining using antibodies against mice VE-cadherin/CD31. Red: VE-cadherin/CD31; Blue: Nucleus; Yellow arrow: Absence of VE-cadherin in the vascular endothelium of the liver section.

CD31 and VE-cadherin play key roles in intercellular connection and vascular permeability. Disturbed expression of them could lead to disrupted junction and increased permeability of endothelium (14, 21) which contributes to adhesion and migration of inflammatory cells. Immunofluorescence staining showed that CD31 and VE-cadherin expression were discontinuous and the continuity of liver blood vessels of the mice were interrupted in GVHD group compared with BMT group (**Figures 4**, **5**). CD31/VE-cadherin expression were also reduced at colon mucosa and lamina propria in GVHD group compared with BMT group (**Supplemental Figure 2**, **Figure 5**). A large number of CD3^+^ T cells were seen around the expansive blood vessels in the liver of GVHD mice on day 21 post transplantation. It suggested that enhanced immune response in the liver blood vessels of GVHD mice. Interestingly, infusion of EPC significantly reduced the number of CD3^+^ T cells around the liver vessels and the vascular continuity of the liver blood vessels was significantly improved after EPC infusion (**Figure 4**). Additionally, infusion of EPC reversed partly CD31/VE-cadherin expression on day 21 post transplantation (**Figure 5**). It was indicated that EPC can repair damaged blood vessels in liver and colon and enhance the stability and integrity of blood vessels.

### Infusion of EPC attenuates abnormal activation of damaged endothelium

**Figure 6:**
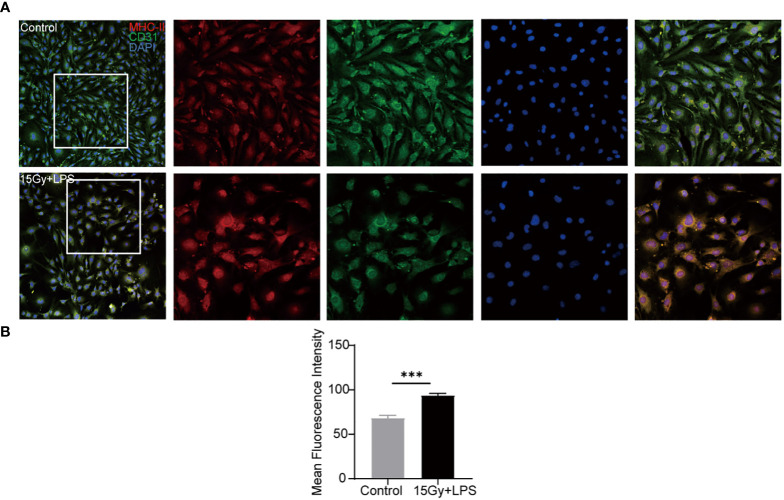
Changes in the expression of MHC-II molecule after endothelial cell injury. Endothelial cells were irradiated (15Gy) and stimulated with LPS (100 ng/ml) for 24 hours followed by immunofluorescence staining with anti- mouse MHC-II molecule and anti- mouse CD31 antibodies. Red: MHC-II molecule, green: CD31, blue: nucleus. **(A)** Image J software analyzed the mean fluorescence intensity of MHC-II molecule in cells (n=4, ^***^ *P*<0.001) **(B)**.

In order to verify the abnormal activation of endothelial cells in aGVHD mice, endothelial cells were subjected to irradiation injury and LPS stimulation and analyzed. The results showed that the mean fluorescence intensity of endothelial cells expressing MHC-II molecule in the injuried group was significantly elevated (P < 0.001) compared with the control group (**Figure 6**), indicating that endothelial cells highly expressed MHC-II molecule with antigen presenting ability when being injured.

**Figure 7:**
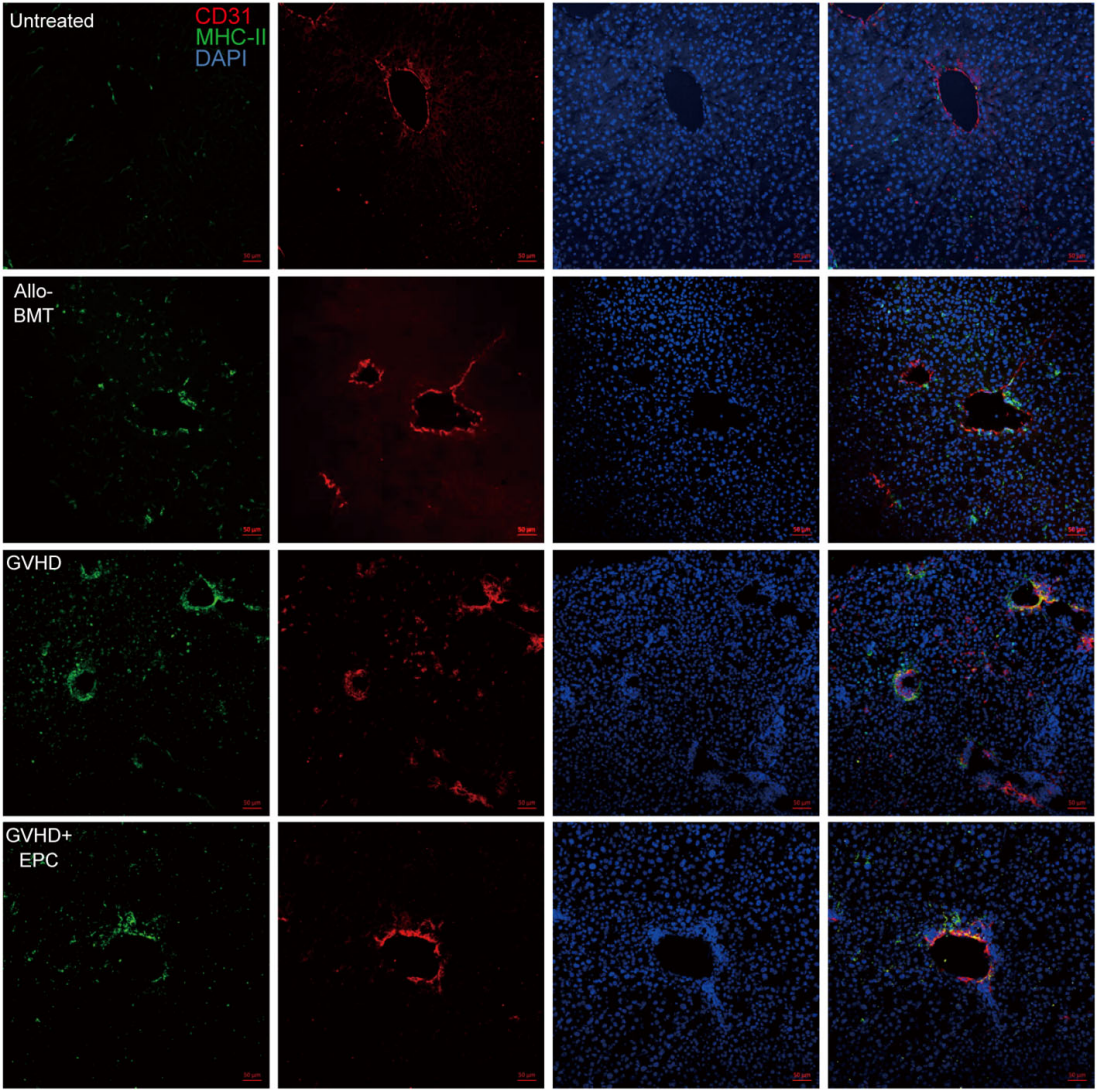
MHC-II molecule expression related to endothelium in the liver. On day 21 after transplantation, livers of each group of mice were collected for dehydration, embedding, and frozen section followed by immunofluorescence staining using antibodies against mice MHC-II molecule and CD31. red: CD31-labeled vascular endothelium; green: MHC-II molecule; blue: Nucleus.

*In vivo*, there were MHC-II^+^ cells in the normal liver blood vessels, which are considered to be Kupffer cells located close to the liver sinusoids. In addition to the Kupffer cells, there were also a small number of MHC-II^+^ cells random distributing in the liver section of allo-BMT group. However, a larger number of MHC-II^+^ cells were observed in the liver tissues of GVHD group. Furthermore, the double positive linear signals of CD31 and MHC-II molecule was also elevated (**Figure 7**). This indicates that vascular endothelial cells of GVHD group expressed MHC-II molecule and acquired the ability to present antigens. Interestingly, MHC-II molecule expression decreased in the liver of GVHD+EPC group, which were consistent with the result *in vitro*. This data shows that Infusion of EPC could downregulate the MHC-II molecule expression on endothelium and reduce pathological activation of endothelial cell.

## Discussion

Acute GVHD is one of the main complications after allogeneic hematopoietic stem cell transplantation, and the occurrence of aGVHD is the result of a complex and comprehensive effect (22). First, radiotherapy and chemotherapy pretreatment lead to tissue damage and antigen exposure. After transplantation, donor T cells recognize the heterotypic antigen presented by APC cells and become activated and migrate to target organs followed by damage and dysfunction of multi organs. The processing of antigens by antigen presenting cells (APCs) plays an important role in aGVHD. The activation and proliferation of donor T cells are the leading cause in the development of aGVHD which results to serious tissue damage (23). Once aGVHD occurs, first-line treatment, that is high-dose steroid hormones, is applied to relief symptoms. However, nearly half of the patients would turn into steroid-refractory graft-versus-host disease (srGVHD) (24). For this reason, exploring new prevention and treatment strategies for GVHD and finding new therapeutic targets have important clinical and practical significance.

Studies have shown that endothelial injury is a potential cause of refractory GVHD. In srGVHD, sTM (serum coagulation factor) levels and the angiopoietin-2 (ANG2)/vascular endothelial growth factor (VEGF) ratio are increased significantly, which will cause endothelial cell damage (25, 26). Our current study found that in aGVHD, T cell infiltration was increased significantly around the vascular endothelium, indicating the endothelial damage which is caused by the infiltration of a large number of T cells. In this process, vascular endothelial damage is inevitable. Endothelial injury destroys vascular leakage and permeability and permits leukocyte adhesion and migration, which could aggravate inflammation and lead to edema, vascular occlusion, thrombosis, and tissue and organ dysfunction (27). EPC is considered to play an important role in repairing endothelial damage (28). In our present studies, we had found that infusion of bone marrow derived EPC could ameliorate endothelial cells injury in the liver on d5, d10, d15 and d20 post hematopoietic stem cell transplantation (14). We found furtherly that GFP^+^ cells survived even till 4 weeks post infusion which suggested bone marrow derived EPC promised regenerative properties. After infusion of EPC, T cell infiltration in aGVHD was significantly reduced. EPC may enhance the stability of blood vessels by repairing damaged vascular endothelium, reduce blood vessel permeability and T cell diapedesis (17). EPCs have also been successfully isolated from tissue resident vascular progenitors, such as fat tissue, but whether EPC from different sources or cell culture technologies have similar effect on aGVHD need extensive studies.

VE-Cadherin is a vascular endothelial cadherin, an endothelial-specific adhesion protein, which also plays an important role in the endothelial barrier (29, 30). Our experiment found that the VE-cadherin of the endothelium in aGVHD was significantly decreased compared with untreated group and allo-BMT group. The decrease of VE-cadherin would cause the destruction of endothelial permeability, which might be a critical contributing factor to subendothelial migration of a large number of T cells. However, infusion of EPC can alleviate the loss of VE-cadherin in the injured endothelium, which is beneficial to endothelial integrity. Therefore, EPC is a prospective cytotherapy to repair endothelium in aGVHD.

Studies have found that in cerebral malaria, brain endothelial cells can present antigens and stimulate T cells to promote the activation of the effector CD4^+^ T cell response. Endothelial cells can act as “semi-professional APC”, can be recognized by TCR, and promote the activation and proliferation of T cells (31, 32). Antigen-presenting cells are the key to T cell activation and proliferation. Our study found that endothelial cells in the tissue can be activated to express MHC-II molecule in aGVHD, thus obtain the function of presenting antigen, which may play a role in the pathogenesis of aGVHD. Studies have shown that there are several molecules that promote the activation of endothelial cell in the serum of GVHD patients (33). For example, interferon-gamma can induce the expression of MHC-II molecule on ECs, leading to activation and proliferation of T cells (34), which is consistent with our results. Infusion of EPCs repairs the damage of endothelium and downregulates the expression of MHC-II molecule in ECs. MHC-II molecule presentation in aGVHD are regulated by myeloid cell and cytokines, it need further exploration about the mechanism of MHC-II molecule in ECs regulated by EPC in aGVHD. In addition, we found that infusion of EPC modulates effector T cell subsets which might contribute to alleviate aGVHD, but the mechanism remains unclear.

Our previous studies have shown that the number of peripheral circulating EPCs in aGVHD increased, indicating that after endothelial injury, the mobilization of EPCs in the body has initiated the endothelial repair process (35). After EPC infusion, it can engraft to the bone marrow and other damaged organs (17). Bone marrow-derived EPC can stimulate angiogenesis and reduce tissue ischemia caused by coronary heart disease (CAD) and peripheral artery disease (PAD) (36, 37). It has been reported that in ischemic retinopathy, EPCs can interact closely with endothelial cells through adhesion and tight junctions to integrate the retinal vascular network (38). Infusion of EPCs can promote neovascularization through the secretion of pro-angiogenic cytokines and growth factors such as IL-8 in critical limb ischemia. And intravascular perfusion of EPCs can improve the prognosis of patients with acute myocardial infarction (AMI) (39). EPCs do not only play a vital role in maintaining endothelial integrity but also can mobilize from bone marrow or exogenously transplantation to ischemic tissues to promote endothelial repair and neovasculogenesis (36). It is need to further explore the mechanism of EPC repairing damaged endothelium in aGVHD.

In conclusion, infusion of bone marrow-derived EPC to aGVHD mice can mobilize to affected endothelium to reduce T cell diapedesis and endothelial activation, alleviate the loss of VE-cadherin in endothelial cell and repair damaged endothelium which contributes to promote endothelial stability. Importantly, EPC infusion can reduce aGVHD severity, the clinical and pathological scores, and prolong survival time of mice. Our study indicates that EPC infusion combined with bone marrow transplantation might be a preventive and therapeutic strategy for aGVHD.

## Data availability statement

The original contributions presented in the study are included in the article/**Supplementary Material**. Further inquiries can be directed to the corresponding authors.

## Ethics statement

The animal study was reviewed and approved by the Animal Ethics Committee of Xuzhou Medical University.

## Author contributions

WW and YY contributed equally to this study. WW performed the experiments, analyzed the data, and wrote the manuscript. YY, YD, ZX, KY, SA, YL and KX carried out parts of the experiments and collected the data. JQ, WJ, and LZ designed the study, analyzed and interpreted the data, and revised the manuscript. All authors contributed to the article and approved the submitted version.
