# Supplementary figures and images for: EPC infusion ameliorates acute graft-versus-host disease-related endothelial injury after allogeneic bone marrow transplantation
Source: Front Immunol. 2022 Dec 14;13:1019657. doi: 10.3389/fimmu.2022.1019657 (PMC9795844; doi:10.3389/fimmu.2022.1019657)

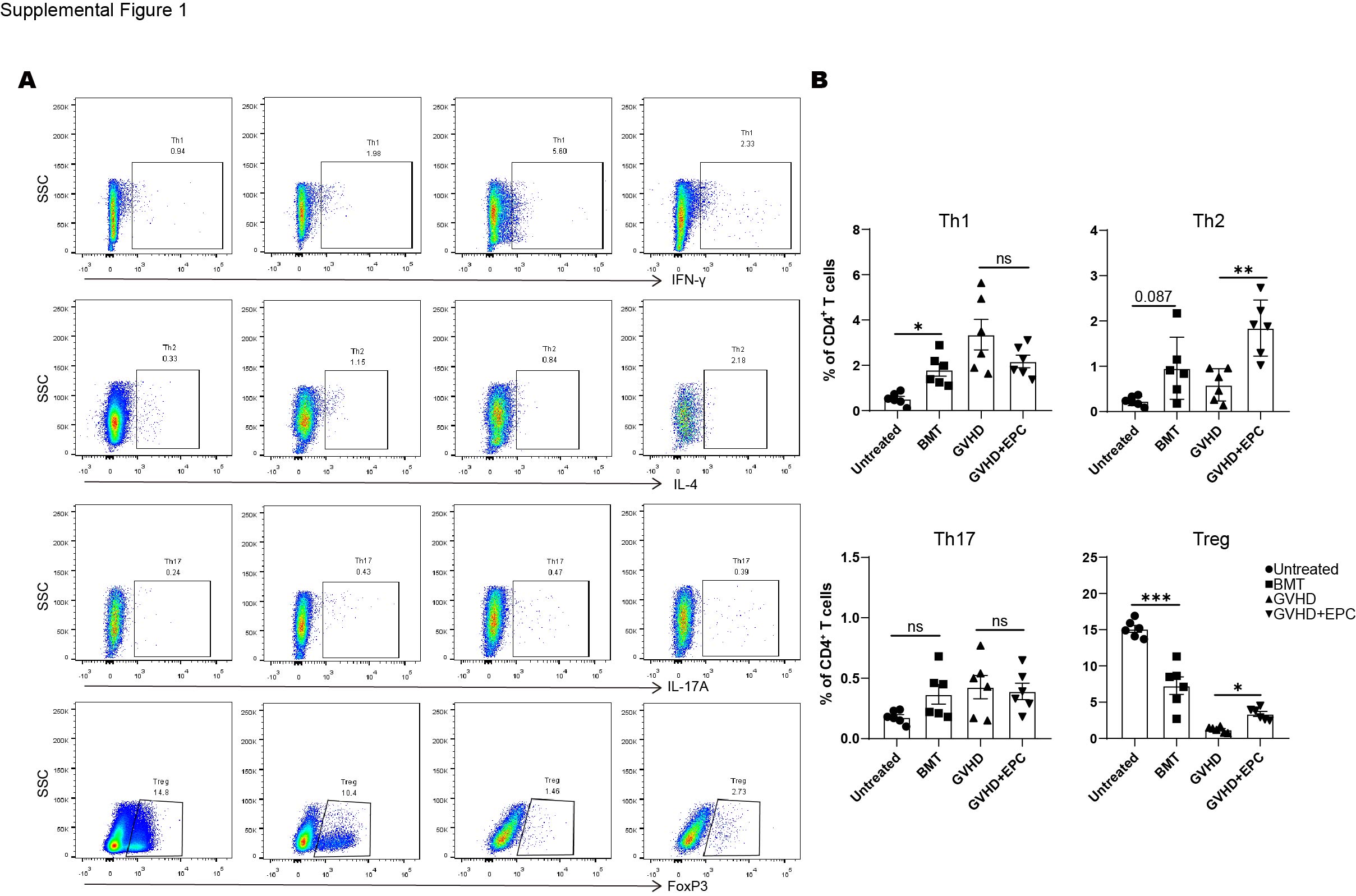

Supplement: Supplementary file 1 [file Image_1.jpg]

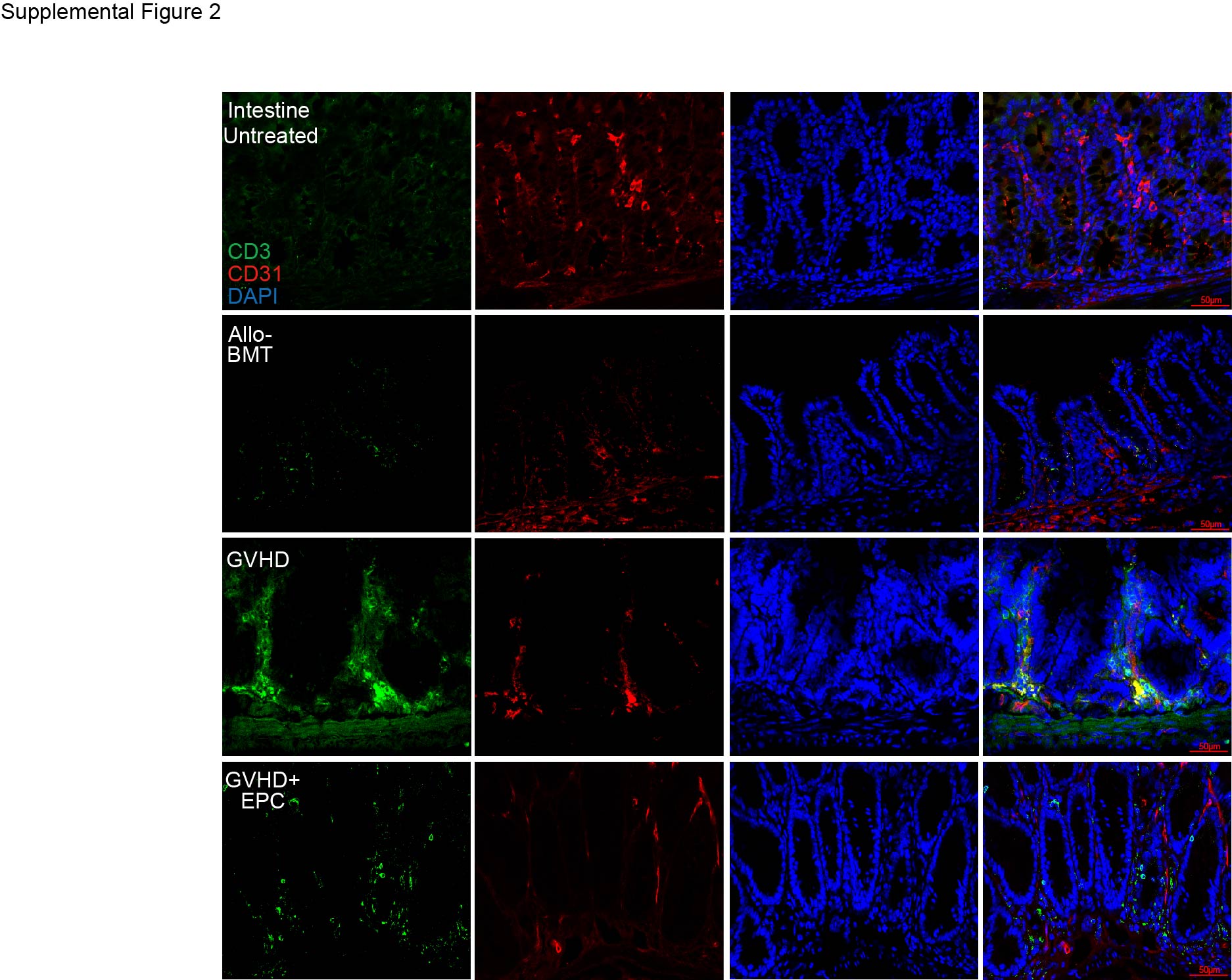

Supplement: Supplementary file 2 [file Image_2.jpg]
